# Supplementary material for: Role and mechanism of NCAPD3 in promoting malignant behaviors in gastric cancer
Source: Front Pharmacol. 2024 Apr 22;15:1341039. doi: 10.3389/fphar.2024.1341039 (PMC11070777; doi:10.3389/fphar.2024.1341039)
Supplement: Supplementary file 11 [file DataSheet2.ZIP › GSEA/GO_CC/my_analysis.Gsea.1599461408869/GO_MITOCHONDRIAL_MATRIX.html]

Details for gene set GO\_MITOCHONDRIAL\_MATRIX[GSEA]

|  || Dataset | filtered\_dataset.sample\_info.cls#WT\_versus\_NCAPD3\_MUT |
| Phenotype | sample\_info.cls#WT\_versus\_NCAPD3\_MUT |
| Upregulated in class | WT |
| GeneSet | GO\_MITOCHONDRIAL\_MATRIX |
| Enrichment Score (ES) | 0.24949028 |
| Normalized Enrichment Score (NES) | 1.4146643 |
| Nominal p-value | 0.08626198 |
| FDR q-value | 0.6377949 |
| FWER p-Value | 0.997 |
Table: GSEA Results Summary

  

Fig 1: Enrichment plot: GO\_MITOCHONDRIAL\_MATRIX      
 Profile of the Running ES Score & Positions of GeneSet Members on the Rank Ordered List

  

| SYMBOL | TITLE | RANK IN GENE LIST | RANK METRIC SCORE | RUNNING ES | CORE ENRICHMENT || 1 | 117145 | THEM4 | 65 | 0.860 | -0.0114 | Yes |
| 2 | 55794 | DDX28 | 68 | 0.856 | 0.0232 | Yes |
| 3 | 2926 | GRSF1 | 75 | 0.849 | 0.0546 | Yes |
| 4 | 55006 | TRMT61B | 101 | 0.782 | 0.0693 | Yes |
| 5 | 92935 | MARS2 | 143 | 0.741 | 0.0705 | Yes |
| 6 | 23590 | PDSS1 | 175 | 0.701 | 0.0773 | Yes |
| 7 | 54516 | MTRF1L | 187 | 0.687 | 0.0982 | Yes |
| 8 | 79922 | MRM1 | 209 | 0.669 | 0.1110 | Yes |
| 9 | 51250 | C6orf203 | 230 | 0.650 | 0.1238 | Yes |
| 10 | 91574 | C12orf65 | 238 | 0.646 | 0.1459 | Yes |
| 11 | 25973 | PARS2 | 266 | 0.620 | 0.1522 | Yes |
| 12 | 81889 | FAHD1 | 299 | 0.592 | 0.1537 | Yes |
| 13 | 36 | ACADSB | 302 | 0.589 | 0.1771 | Yes |
| 14 | 92399 | MRRF | 347 | 0.569 | 0.1689 | Yes |
| 15 | 166785 | MMAA | 369 | 0.553 | 0.1768 | Yes |
| 16 | 326625 | MMAB | 371 | 0.552 | 0.1993 | Yes |
| 17 | 11019 | LIAS | 409 | 0.533 | 0.1947 | Yes |
| 18 | 84274 | COQ5 | 419 | 0.523 | 0.2101 | Yes |
| 19 | 54931 | TRMT10C | 446 | 0.507 | 0.2125 | Yes |
| 20 | 4191 | MDH2 | 494 | 0.476 | 0.1981 | Yes |
| 21 | 6742 | SSBP1 | 501 | 0.472 | 0.2136 | Yes |
| 22 | 34 | ACADM | 530 | 0.457 | 0.2123 | Yes |
| 23 | 56652 | C10orf2 | 531 | 0.457 | 0.2316 | Yes |
| 24 | 78988 | MRPL57 | 557 | 0.441 | 0.2319 | Yes |
| 25 | 27349 | MCAT | 574 | 0.436 | 0.2386 | Yes |
| 26 | 26164 | MTG2 | 585 | 0.433 | 0.2495 | Yes |
| 27 | 219 | ALDH1B1 | 638 | 0.405 | 0.2285 | No |
| 28 | 55157 | DARS2 | 686 | 0.385 | 0.2103 | No |
| 29 | 6182 | MRPL12 | 729 | 0.359 | 0.1947 | No |
| 30 | 64087 | MCCC2 | 747 | 0.352 | 0.1970 | No |
| 31 | 8803 | SUCLA2 | 767 | 0.340 | 0.1975 | No |
| 32 | 55178 | RNMTL1 | 771 | 0.337 | 0.2095 | No |
| 33 | 9238 | TBRG4 | 786 | 0.330 | 0.2131 | No |
| 34 | 79590 | MRPL24 | 800 | 0.320 | 0.2171 | No |
| 35 | 29960 | FTSJ2 | 805 | 0.319 | 0.2276 | No |
| 36 | 4285 | MIPEP | 815 | 0.307 | 0.2340 | No |
| 37 | 51373 | MRPS17 | 817 | 0.306 | 0.2461 | No |
| 38 | 6183 | MRPS12 | 838 | 0.280 | 0.2433 | No |
| 39 | 2746 | GLUD1 | 854 | -0.255 | 0.2431 | No |
| 40 | 10797 | MTHFD2 | 911 | -0.338 | 0.2163 | No |
| 41 | 384 | ARG2 | 945 | -0.366 | 0.2075 | No |
| 42 | 5163 | PDK1 | 982 | -0.394 | 0.1978 | No |
| 43 | 25902 | MTHFD1L | 986 | -0.395 | 0.2122 | No |
| 44 | 84706 | GPT2 | 1076 | -0.460 | 0.1664 | No |
| 45 | 5106 | PCK2 | 1252 | -0.640 | 0.0652 | No |
| 46 | 3329 | HSPD1 | 1402 | -1.183 | 0.0059 | No |
Table: GSEA details [plain text format]

  

Fig 2: GO\_MITOCHONDRIAL\_MATRIX      
 Blue-Pink O' Gram in the Space of the Analyzed GeneSet

  

Fig 3: GO\_MITOCHONDRIAL\_MATRIX: Random ES distribution      
 Gene set null distribution of ES for **GO\_MITOCHONDRIAL\_MATRIX**
